# Effectiveness of brief psychological interventions for suicidal presentations: a systematic review

**DOI:** 10.1186/s12888-018-1663-5

**Published:** 2018-05-03

**Authors:** Rose McCabe, Ruth Garside, Amy Backhouse, Penny Xanthopoulou

**Affiliations:** 1 University of Exeter Medical School, Heavitree Road, Exeter, EX1 2LU UK; 2 European Centre for Environment and Human Health, Knowledge Spa, Royal Cornwall Hospital, Truro, TR1 3HD UK

**Keywords:** Suicide, Suicidal ideation, Systematic review, Controlled studies, Effective communication

## Abstract

**Background:**

Every year, more than 800,000 people worldwide die by suicide. The aim of this study was to conduct a systematic review of the effectiveness of brief psychological interventions in addressing suicidal thoughts and behaviour in healthcare settings.

**Methods:**

Following PRISMA guidelines, systematic searches were conducted in MEDLINE, CINAHL, EMBASE, the Cochrane Central Register of Controlled Trials and PsycINFO databases. A predefined search strategy was used. Two independent reviewers screened titles and abstracts followed by full texts against predefined inclusion criteria. Backward and forward citation tracking of included papers was conducted. Quality appraisal was conducted using the Cochrane Risk of Bias Tool for Randomized Controlled Trials and the CASP tool for randomised controlled trials. The small number and heterogeneity of studies did not allow for meta-analysis to be conducted. A narrative synthesis was conducted.

**Results:**

Four controlled studies of brief psychological interventions were included, conducted in Switzerland, the U.S. and across low and middle-income countries. Three studies were conducted with adults and one with adolescents. All studies were judged to be at low risk of bias. All of the interventions were implemented with patients after attending emergency departments and involved 3412 participants. The main outcomes were suicide, suicide attempts, suicidal ideation, depression and hospitalization. The components of the interventions were early therapeutic engagement, information provision, safety planning and follow-up contact for at least 12 months. The interventions drew to, different degrees, on psychological theory and techniques. Two trials that measured suicidal ideation found no impact. Two studies showed fewer suicide attempts, one showed fewer suicides and one found an effect on depression.

**Conclusions:**

Although the evidence base is small, brief psychological interventions appear to be effective in reducing suicide and suicide attempts. All studies to date have been conducted with people who had attended the ED but the interventions could potentially be adopted for inpatient and other outpatient settings. Early engagement and therapeutic intervention based on psychological theories of suicidal behaviour, sustained in follow-up contacts, may be particularly beneficial.

**Trial registration:**

Systematic review registration: PROSPERO CRD42015025867.

**Electronic supplementary material:**

The online version of this article (10.1186/s12888-018-1663-5) contains supplementary material, which is available to authorized users.

## Background

Suicide is a serious public health concern with more than 800,000 deaths from suicide every year worldwide [1]. This is one suicide every 40 seconds [2]. Suicide prevention is a global public health priority.

Certain groups have a higher risk of suicide. The majority of deaths by suicide (78%) occur in low and middle-income countries. There are also significant gender differences with men more likely to die by suicide (male-to-female ratio 1.7 in 2015) [1]. Younger people are also more likely to die by suicide: 55% of deaths by suicide are among the 15–44 age group with suicide ranked as the second leading cause of death among 15–29 year-olds [1].

Many people who take their own life have been in contact with healthcare professionals in acute hospitals and/or primary or secondary care before they die. In the U.K., 40% of people attended the general emergency department in the year before death, having attempted suicide [3]. Around one in four people who take their own life have been in contact with mental health services the year before death in the U.K. and around one in three in the U.S. [4]. Meanwhile, 45% of people who take their life were seen in primary care the month before death in the U.K. with a similar figure of 47% in the U.S. [4].

Collectively, this is a very high number of face-to-face contacts between healthcare professionals and people who go on to take their own life. Referring patients to specialist services is often not a realistic option because they are not available or where they are available, there is not enough capacity in these services. Specialist treatment is very costly and many patients do not attend or drop out of treatment prematurely [5]. Hence, in routine contacts with people at risk of suicide, there is potential for brief therapeutic interventions. There are longer term psychological interventions (e.g. dialectical behaviour therapy, cognitive behaviour therapy) to address suicide [6, 7] and self-harm [8]. However, it is not clear if limited brief interventions that can be administered in routine frontline encounters where healthcare professionals encounter people at risk of suicide can be effective [9, 10].

Studies in healthcare and other settings (e.g. educational) [11, 12] generally report brief interventions as lasting 1–3 sessions [13, 14]. We focused on brief interventions as they are more likely to be integrated into routine clinical practice without the need for significant additional resources or extensive reconfiguration of existing services. Brief interventions that could be deployed in routine care, rather than referring people to another service, are of particular interest as they could be deployed at scale to improve patient outcome.

### Objectives

The aim of this review was to evaluate the effectiveness of brief psychological interventions to address suicidal thoughts and plans, focusing on two objectives:

1. To identify controlled studies of brief psychological interventions to address suicidal thoughts and plans in healthcare settings.
2. To describe the interventions used by professionals/paraprofessionals that are effective in addressing suicidal thoughts and plans.

## Methods

### Protocol and registration

Approaches to searching, methods of analysis and inclusion criteria were specified in advance and documented in a protocol [15], with some changes made in the course of the study (recorded on PROSPERO: CRD42015025867), relating mainly to the eligibility criteria. The PRISMA standards of quality for reporting meta-analyses [16] were used to plan, conduct and report this review.

### Eligibility criteria

The review included published controlled studies that reported on brief psychological interventions to address suicidal thoughts and plans in healthcare settings.

#### Inclusion criteria

##### Participants

Participants of any age and gender at risk of suicide*.*

##### Interventions

Brief interventions delivered in any healthcare setting to the specified population:

- Interactions between professionals/paraprofessionals (e.g. lay mental health workers, nursing assistants, educators, volunteers) and patients
- Addressing suicidal thoughts and plans
- Two-way communication (i.e. not one-way communication in the form of letters/postcards/text messages or exclusively self-guided questionnaires/instruments) between at least one professional/paraprofessional and one patient; other people can be present
- Focus on suicidal thoughts and plans rather than diagnostic conditions, e.g. depression, anxiety, borderline personality disorder
- Focus on routine clinical encounters
- Brief interventions, defined as up to three sessions delivered in/soon after presenting episode, which can be supplemented by further follow-up contact

##### Comparator

Any comparison or no comparator/usual care.

##### Outcome measures

Primary outcome was suicidal ideation, using any measure. Other outcomes included: Identification of suicide risk, suicide attempts, suicide, hope, patient distress and depression. Suicidal ideation is defined according to Beck’s ‘Scale for Suicide Ideation’ [17] as the intensity of current conscious suicidal intent, examining various dimensions of self-destructive thoughts or wishes.

##### Types of studies

No restrictions were placed on study location or publication date of included studies. We included cluster randomised controlled trials, randomised controlled trials, controlled before-and-after studies and controlled pre-test/post-test designs. We excluded non-controlled studies.

##### Exclusion criteria

Assisted suicide; Self-harm without intent to die, i.e., direct, deliberate destruction of one’ s own body tissue in the absence of intent to die, which differs from suicide attempts with respect to intent, lethality, chronicity, methods, cognitions, reactions, aftermath, demographics and prevalence [18].

### Search and information sources

Database searches were conducted from date of inception to June 2015, and updated in August 2016 and in April 2017. The following databases were searched: MEDLINE in Process (Ovid), PsycINFO (Ovid), EMBASE (Ovid), The Cochrane Central Register of Controlled Trials (CENTRAL) (Wiley Online Library) and CINHAL (EBSCO). Trial registers (ISRCTN registry, ClinicalTrials.gov) were searched for published and ongoing trials, references of previous systematic reviews were searched and experts in the field were contacted in order to identify any new studies.

The search strategy is presented in Additional file 1. Suicide, study design and communication/interaction terms were combined using Boolean logic (AND, OR) and specific tested filters were used for study design (The InterTASC Information Specialists’ Sub-Group filters). Medical Subject Heading (MeSH) terms were also used. EndNote X7.0.2 software was used to manage searches and references.

### Study selection

Search results were exported to EndNote and duplicates were automatically identified and removed. Records that were not removed automatically we identified and removed by hand. Two independent reviewers (PX, RM/AB) were involved in screening all titles and abstracts, full paper screening, quality appraisal and assessment of risk of bias of included studies. Disagreements or uncertainties were discussed in meetings and email correspondence between all authors.

### Data extraction

We developed a data extraction form based on the Cochrane Risk of Bias Tool for Randomized Controlled Trials, which we modified to reflect the diversity of included studies. The extraction form was piloted (RM, AB, PX) before being finalised. Data was extracted by one author (RM/PX) and checked by another (RM, AB, PX). The authors of three included studies were contacted to obtain additional data, trial protocols and further detail on the relevant intervention. Additional information was also obtained from other publications reporting on the study [19].

### Risk of bias

Risk of bias was assessed using the Cochrane Risk of Bias Tool for Randomized Controlled Trials on 6 criteria. Each criterion was rated as low, medium or high. Using these ratings, we generated an overall risk of bias score by scoring the ratings on the first 5 criteria: sequence generation, allocation concealment, blinding, incomplete outcome data and selection bias. A score of 3 was allocated to a ‘low’ risk, a score of 2 was allocated to ‘medium’ risk and a score of 1 was allocated to ‘high’ risk. The total score could range from 5 to 15, with a higher score indicating lower risk of bias.

Study quality was also assessed using the CASP (Critical Appraisal Skills Programme) for randomised controlled trials checklist [20]. Two raters independently assessed the risk of bias for each study (PX and RM/AB). The individual items on the score sheets were then checked by three authors (RM, AB, PX) in an inter-reviewer discussion where disagreements were resolved.

### Analysis

The studies were too heterogeneous to combine in a meta-analysis, in terms of the outcomes they measured. Hence, a narrative synthesis [21] was conducted. This involved developing a preliminary synthesis, focusing on the outcomes, interventions and heterogeneity across the studies, followed by iteratively exploring relationships in the data, contexts of the interventions and mechanisms for change, using visual representations (tables) [21]. Where not available, relative risk was calculated using the MEDCALC relative risk statistical calculator (https://www.medcalc.org/calc/relative_risk.php).

## Results

### Study selection

**Fig. 1:**
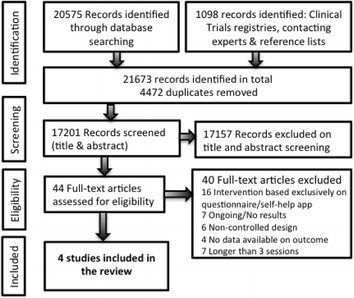
PRISMA Flow diagram of the study selection and screening process

After removing duplicates, a total of 17,201 titles and abstracts were identified and screened (Fig. 1). Of these, 44 full-text articles were assessed for eligibility. Forty full-text articles were excluded studies due to a lack of control in the study design, no data available on treatment outcome, interventions were exclusively based on questionnaires or longer than three sessions. Four studies met the inclusion criteria and were included in the review.

The included studies encompassed two RCTs, one pilot RCT and a quasi-experimental study. All reported on interventions in the emergency department setting. The non-randomised controlled study used an interrupted time series design [22] and the three RCTs involved individual patient randomization [23–25]. The interventions were compared to treatment as usual (TAU) [23] and enhanced TAU [22, 24, 25].

### Risk of bias

**Fig. 2:**
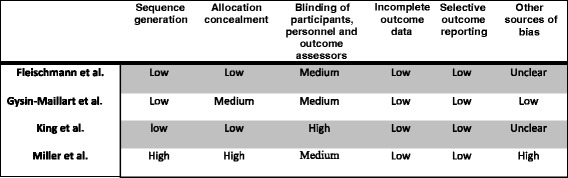
Risk of bias assessment (Cochrane Risk of Bias Tool for Randomized Controlled Trials)

The risk of bias assessment, using Cochrane Risk of Bias Tool for Randomized Controlled Trials, is presented in Fig. 2. The overall score for each study (see Methods section for scoring) was: Fleischmann [23] 14 out 15, Gysin-Maillart [24] 13 out of 15, King [25] 13 out 15, and Miller [22] 10 out 15.

Three studies were of high quality. High/medium risk of bias was reported for blinding professionals across all studies, however, it would not have been possible to blind professionals as they were delivering the interventions. One study [22] presented medium risk of bias, where lower scores related to not using randomization to allocate interventions to participants (the study employed an interrupted time series design [26]).

Studies rated high in the CASP for randomised controlled trials checklist (results are presented in Additional file 2).

### Characteristics of participants and outcomes

In total, the studies included 3412 participants (range 49–1867). Three studies included adult suicide attempters [22–24] and one [25] focused on adolescents with suicide risk factors (e.g. depression and alcohol misuse). Study characteristics are presented in Table 1.

**Table 1:** Summary characteristics of included studies

	Participants	Nature of suicide risk	Study Design	Setting	Intervention	Control	Pre-intervention patient measures	Post-intervention patient measures	Outcomes	Follow up period
Fleischmann et al 2008 [23] 5 sites (Brazil; India; Sri Lanka; Iran; and China)	1867 Adults 57% female, median age 23 years	Patients who have attempted suicide	RCT Individual randomization	Emergency care settings	One-hour individual information session & periodic follow-up contacts after discharge for 18 months	TAU (as per norms in the respective EDs)	Questionnaire based on the European Parasuicide Study Interview Schedule (EPSIS) and adapted to each site	One-page questionnaire: if patient still alive; if not cause of death; if yes any further suicide attempts; how the patient felt; needs for support	Primary: Completed suicide	18 months
Gysin-Maillart et al 2016 [24] Switzerland	120 Adults 55% female, Mean age 37.8 years	Patients admitted to the ED who attempted suicide	RCT Individual randomization	Emergency department	3 face-to-face therapy sessions supplemented by regular, personalized letters to the participants for 24 months	Enhanced TAU: TAU (inpatient, day patient, and individual outpatient care as considered necessary by the clinicians in charge) and one clinical interview	Suicide Status Form (SSF-III) and 33-item questionnaire to collect sociodemographic, health and suicidal behaviour data	Penn Helping Alliance Questionnaire; Beck Depression Inventory; Beck Scale for Suicide Ideation	Primary: Repeat suicide attempts Secondary: Suicidal ideation, Depression, Health-care utilisation.	2 years
King et al 2015 [25] United States	49 Adolescents 65% female 14–19 years,	Patients with suicide risk factors	Pilot RCT Individual randomization	Emergency department	Personalized feedback, adapted motivational interview and follow-up note	Enhanced TAU (basic mental health resources: crisis card, written information about depression, suicide risk, firearm safety and local mental health services)	2 questions based on the Columbia-Suicide Severity Rating Scale; 15-item Suicidal Ideation Questionnaire – Junior (SIQ-JR); Reynolds Adolescent Depression Scale; Alcohol Use Disorders Identification Test; Beck Hopelessness Scale	Two questions adapted from the Columbia-Suicide Severity Rating Scale; Reynolds Adolescent Depression Scale; The Beck Hopelessness Scale; The Alcohol Use Disorders Identification Test; Motivational interviewing	Depression, hopelessness, suicidal ideation and alcohol use.	2 months
Miller et al 2017 [22] United States	1376 Adults 55.9% female median age 37	Patients attending ED with suicide attempt or ideation in previous week	Interrupted time series design	Emergency department	1. Secondary suicide risk screening 2. Self-administered safety plan & information provided by nurses 3. Telephone follow-up to patients and a significant other	TAU (usual care at each site) and contacts for 1 year	None	1. Telephone interviews using the Columbia Suicide Severity Rating Scale 2. Medical records	Suicide attempts, Suicide composite: occurrence of suicide, suicide attempt, interrupted/ aborted attempts & suicide preparatory acts	1 year

As there were only four studies that differed in what outcomes they assessed and when these outcomes were assessed (2, 12, 18 and 24 months), a meta-analysis was not appropriate. One study was conducted across 5 countries and the included paper reports results across all 5 sites. Separate results for one of the sites (Iran) are reported elsewhere [27], however we did not include this study due to overlapping data.

### Characteristics of interventions

The interventions focused on engagement, safety planning, information and follow-up contact after discharge from the emergency department. The duration of the interventions ranged from 12 to 24 months.

The four interventions in the included studies were:

- brief intervention and contact (BIC) [23]
- the attempted suicide short intervention program (ASSIP) [24]
- teen options for change (TOC) [25]
- Safety Assessment and Follow-up Telephone Intervention (SAFTI) [22]

A summary of the interventions is presented in Table 2. The three larger studies [22–24] used 1–3 individual sessions soon after discharge from the ED and follow-up contacts over 18, 24 and 12 months respectively. The interventions varied according to when the intervention starts, whether patients are seen soon after discharge from the ED and then how often they are contacted during the follow-up period.

**Table 2:** Description of interventions

	Theoretical foundation	Characteristics of professionals delivering the intervention	Professional training in intervention	When was the intervention started	Intervention Components	No. & length of initial session/s	No., mode & frequency of follow up contacts	Who delivers contact/s in the ED	Who delivers contact/s after ED	Content of follow-up contacts	Intervention completion
Fleischmann et al 2008 [23]	Not described	Trained psychiatrists, medical doctors, psychologists or psychiatric nurses	Not described	Within 3 days after assessment in ED	1. Information session: information about suicidal behaviour as a sign of psychological and/or social distress, risk and protective factors, basic epidemiology, repetition, alternatives to suicidal behaviours, and referral options. 2. Follow up contacts over 18 months	One 1-hr individual information session	9 telephone /face-to-face contacts at 1, 2, 4, 7 and 11 week(s), and 4, 6,12 and 18 months)	Trained psychiatrists doctors, psychologists or psychiatric nurses	Doctor, nurse, psychologist	Phone calls or visits	91% received the full intervention
Gysin-Maillart et al 2015 [24]	Action Theory, Cognitive Behaviour Therapy, and Attachment Theory.	Four therapists: one psychiatrist, one psychologist experienced in clinical suicide prevention and two psychological therapists	1-week ASSIP training. Adherence: peer reviews and supervision	Soon after assessment in ED	1. Session 1: narrative interview - patients were asked to tell their personal stories about how they had reached the point of attempting suicide 2. Session 2: Watch session 1 video-recording & psychoeducative handout-homework 3. Session 3: Discussion & case conceptualization: goals, warning signs, and safety strategies. Written case conceptualization, safety strategies & leaflet 4. 6 follow-up letters	Three 60–90 min sessions on a weekly basis	6 letters over 24 months: every 3 months in the first year and every 6 months in the second year	Clinicians and therapists	Clinicians and therapists	Semi-standardized letters –to maintain the therapeutic relationship & reinforce safety strategy	93% completed the intervention at 24 months (95% at 12 months)
King et al 2015 [25]	Motivational Interviewing, Self Determination Theory, Theory of Health Behavior, and Theory of Planned Behavior	Three licensed Social Workers	Min 40 Hours - conducted by a member of the Motivational Interviewing Trainers’ Network	After initial emergency room visit	1. Individual AMI: personalized feedback to the teen, to explore ambivalence, build discrepancy, enhance teen’s problem importance and readiness to change 2. Family AMI: with parent/guardian to develop Personalized Action Plan Form, provide supplemental resource materials 3. Follow-up letter & telephone call	One individual 30–45 min session One family 15–20 min session	Handwritten follow-up note and a telephone check-in two to five days after ED visit to support and facilitate action plan implementation	Study therapists	Study therapists	Personalized follow up note & telephone check-in: Half receive telephone follow-up only.	85% received the full intervention
Miller et al 2017 [22]	Not described	ED physicians & nurses	Detailed manual of procedures, meetings and monthly teleconference to receive training updates, and problem solve	In the ED	1. Secondary suicide risk screening by ED physician following an initial positive screen 2. self-administered safety plan and information to patients by nursing staff 3. follow-up telephone calls	Not described	Up to 7 brief (10–20 min) telephone calls to the patient and up to 4 calls to a significant other, at 6, 12, 24, 36, and 52 weeks	ED physicians and nursing staff	10 advisors: 6 PhD psychologists, 3 psychology fellows, and 1 masters-level counselor	Case management, individual psychotherapy and significant other involvement following Coping Long Term with Active Suicide (CLASP)-ED protocol	1. Secondary suicide risk screening: 89.4% 2. Safety plan: 37.4% 3. Follow-up: 60.8% patients completed at least 1 phone call: of these median number 6 calls (range 2–7). 19.9% patients had a significant other who completed at least 1 call: of these median number of 4 calls (range 3–4)

Gysin-Maillart [24] implemented a therapeutic intervention focused on engaging the person in a narrative interview about the suicidal crisis in a first session soon after the ED attendance. This then progressed to case conceptualization and individualized safety planning in another 2 sessions. Then, patients were contacted via letter for 24 months, every 3 months in the first year and every 6 months in the second year. Fleischmann [23] implemented a single information session to understand and manage suicidal behaviour followed by up to 9 phone calls or visits over 18 months. In the trial by Miller et al. [22], the intervention consisted of secondary suicide screening, information provided by nurses, a self-administered safety plan and up to 7 brief (10–20 min) calls to the patient and up to 4 calls to a significant other.

#### Theoretical rationale and aims of the interventions

The interventions, to varying degrees, focus on informing people about suicidal behaviour, helping people to become aware of problems/vulnerability/events linked to the behaviour, exploring ambivalence and motivating people to engage in safety planning and help-seeking, problem solving and developing practical strategies to manage future suicidal crises along with signposting to helplines/professionals.

Two interventions (BIC, ASSIP) foreground the role of the relationship: BIC follow-up contacts aim to give patients a feeling of being seen and heard by someone. ASSIP aims to establish an early therapeutic alliance to maximise engagement in treatment, with the follow-up contact reinforcing the relationship.

While all of the interventions comprise information, safety planning and follow-up contact, they varied in the extent to which they used psychological theories and techniques. Gysin-Maillart’s and King’s interventions were based more on psychological theories (i.e. action theory and theory of health behaviour) and techniques than Fleischmann and Miller.

#### Completion of the intervention

Completion of the intervention ranged from 60.8% to 93% across studies. In the Fleischmann trial, it appears that 91% received the intervention. In the Gysin-Maillart trial, 93% completed the intervention. 85% of patients in the King trial received the full intervention. Miller et al. reported that 60.8% received at least part of the intervention (i.e. 1 telephone call).

#### Completion of outcome assessments

Fleischmann reported a 9% loss to follow-up at 18 months. Gysin-Maillart reported a 14% loss to follow-up at 24 months. King reported low loses (6%), however this was for a very short follow up period of 2 months. Miller et al. reported that assessment of suicide attempts was conducted for all participants during the 52-week follow-up period, whereas 20% (1089 of 1376 enrolled participants) did not have a suicide composite outcome, which was derived from the telephone interview (self-reported) at 52 weeks.

### Effectiveness of interventions

Brief psychological interventions were effective in reducing suicide, suicide attempts and depression (see Table 3). Interventions used a range of methods to measure these outcomes.

**Table 3:** Primary and Secondary Outcomes

	Suicide	Repeat suicide attempts	Suicide composite	Suicidal ideation	Depression	Health-care utilization	Hopelessness	Alcohol Use
Type of outcome	Behavioural	Behavioural	Behavioural	Self-rated	Self-rated	Self-report & records	Self-rated	Self-rated
Fleischmann et al 2008 [23] *n* = 1867 Risk of bias score^a^: 14/15	Fewer suicides: 0.2% intervention vs. 2.2% control (x^2^ = 13.83; *P* < 0.001) RR = 0.10 (0.02 to 0.45)	n/a	n/a	n/a	n/a	n/a	n/a	n/a
Gysin-Maillart et al 2016 [24] *n* = 120 Risk of bias score: 13/15	n/a	Fewer suicide attempts 8.3% intervention vs. 26.7% control (Wald χ^2^_1_ = 13.1, 95% CI 12.4–13.7, *p* < 0.001) HR 0.17 (0.07–0.46)	n/a	No difference found	No difference found	72% fewer days in hospital after 1 year (ASSIP: 29 d; control group: 105 d; W = 94.5, *p* = 0.038) but not significant at 2 years	n/a	n/a
King et al 2015 [25] *n* = 49 Risk of bias score: 13/15	n/a	n/a	n/a	No difference found	Lower depression intervention Mean (SD) 25.4 (4.7) vs. 30.9 (4.0) control, F = 10.84, df = 1,44; *p* < .01 (Cohen’s d = 1.07; large effect size)	n/a	No difference found	No difference found
Miller et al 2017 [22] *n* = 1376 Risk of bias score: 10/15	n/a	Fewer suicide attempts: TAU, 22.9% (114/497); INT, 18.3% (92/502) RR 0.80 (0.63 to 1.02)	Lower suicide composite: TAU: 48.9% (243/497) INT: 41.4% (208/502) RR 0.85 (0.74 to 0.97)	n/a	n/a	n/a	n/a	n/a

*TAU* treatment as usual, *INT* intervention

^a^Higher score indicates lower risk of bias

#### Suicidal ideation

Two studies found no effect for suicidal ideation [24, 25].

#### Suicide

One trial was effective in reducing suicide over 18 months, with a 90% relative risk reduction in completed suicides [23] (RR = 0.10, 95% CI 0.02 to 0.45, *p* = 0.0025).

#### Suicide attempts

Two studies reported an effect for repeat suicide attempts. Miller [22] reported a relative risk reduction of 20% for the intervention phase (RR 0.80, 95% CI 0.63 to 1.02). Gysin-Maillart [24] reported a mean hazard ratio of 0.17 (95% CI 0.07–0.46), indicating that the ASSIP group had an 83% reduced risk of attempting suicide during the 24-month follow-up period compared to the control group (Wald χ2 1 = 13.1, 95% CI 12.4–13.7, *p* < 0.001). They also conducted an analysis removing those with BPD and found that when individuals with BPD were excluded, the ASSIP group had an 89% lower risk of attempting suicide (mean hazard ratio of 0.11 (95% CI 0.03–0.49)).

Miller [22] also reported an effect for a ‘*suicide composite’* measure (RR 0.85, 95% CI 0.74 to 0.97), which measured 5 types of suicidal behavior: death by suicide, suicide attempt, interrupted or aborted attempts, and suicide preparatory acts.

#### Depression

Of the two studies assessing depression, one study by King [25] found a significant effect, however another by Gysin-Maillart [24] did not. King focused on adolescents over a shorter follow-up period of 2 months while Gysin-Maillart focused on adults (with longer-standing difficulties) over a longer follow-up of 2 years.

#### Healthcare use

Gysin-Maillart [24] found a significant reduction in hospitalization, with 72% fewer days in hospital over 1 year, which was no longer significant after 2 years (*p* = 0.08).

#### Alcohol use and hopelessness

Where assessed [25], no effect was found for alcohol use and hopelessness.

##### Analysis

Miller [22] and Gysin-Maillart [24] used intention to treat analysis. However, Fleischmann [23] did not: they analysed the participants who were not lost to follow-up which corresponded to 91% of the sample. King’s [25] analysis of intervention effect used per protocol, rather than intention to treat, analysis. We cannot tell the direction or magnitude of impact of this, but there was little loss to follow-up (< 10% LTFU).

## Discussion

Four controlled studies of brief psychological interventions to reduce suicidal behaviour and suicide were identified, three with adults and one with adolescents. All of the interventions were implemented with patients who had attended the ED and involved a total of 3412 participants. The interventions had three common components, namely information about/understanding of the suicidal crisis, safety planning and follow-up contact along with different degrees of psychological input. One (out of one study assessing suicide) found fewer suicides [23]. One (out of one study) assessed a ‘suicide composite’ score [22] and found a lower suicide composite score. Two (out of two studies assessing suicide attempts) found fewer suicide attempts [22, 24]: Miller found a small but meaningful difference with a number needed to treat of 22 and Gysin found an 83% reduced risk of attempting suicide during the 24-month follow-up period. Two (out of two) studies measuring suicidal ideation did not show an effect [24, 25]. One (out of two studies measuring depression) found an improvement in depression [25]. One (out of one study measuring hopelessness) found no improvement in hopelessness [25]. One (of one study assessing hospitalisation) found 72% fewer days in hospital after 1 year but no significant difference after 2 years [24]. Hence, there appear to be greater changes in behavioural outcomes than in symptom outcomes, suggesting that patients may still be experiencing suicidal ideation but make fewer suicide attempts and are less likely to die by suicide.

One trial [23] found an effect on suicide, which was conducted across 5 low and middle-income countries. The authors concluded that a brief intervention was likely to have reduced suicide by providing a social support network for people with limited social support in countries with modest infrastructure and financial/human resources. Two trials found an effect on suicide attempts. One was a large trial in the U.S. with 1376 participants [22] and one a small trial in Switzerland with 120 participants [24]: the large trial found a 20% relative risk reduction and the smaller trial with a 83% relative risk reduction. The large trial focused on information provided by nurses and a self-administered safety plan in the ED, followed by 7 telephone calls to the patient and 4 calls to a significant other over one year. Meanwhile, the smaller trial demonstrating the larger effect, focused on 3 face-to-face therapeutic sessions soon after discharge from the ED and follow-up letters over 24 months.

Given the low prevalence of suicide as an outcome, studies in this area use various proxy and composite measures. One study reported on completed suicide, two studies reported on suicide re-attempts and only one study reported healthcare utilisation (i.e. hospitalisation). Suicide attempts were measured using hospital records, however Miller also used telephone interviews to collect information on this outcome, which could address some of the issues of reliability and accuracy of hospital records. This area would benefit from more RCTs with larger populations, that report on completed suicide [6].

What might explain the large effect in the smaller trial? The two trials recruited participants with a similar age range (mean = 37.8 in Gysin’s smaller trial, and median = 37 in Miller’s larger trial) and male-to-female ratio (Gysin 55% female, Miller 56% female). However, the smaller trial [24] was conducted in one ED while the larger trial [22] was conducted across 8 EDs so local championing and fidelity to the intervention may have been stronger in the single site smaller trial. In addition, the smaller trial involved more intensive psychological input with an emphasis on an early therapeutic alliance in face-to-face sessions along with follow-up contact by the same rather than different professionals. A better therapeutic alliance was associated with a lower rate of suicide attempts [28], suggesting that early engagement and therapeutic intervention soon after the ED attendance may be particularly beneficial.

The interventions varied on some important factors, most notably the psychological theories underpinning the intervention, the intensity of and the proposed mechanisms and wider socioeconomic context of the intervention. These were to some extent reflected in when, how and by whom the initial and follow-up contacts were made and what happened in these contacts. The BIC intervention leading to fewer deaths by suicide in low/middle-income countries focused on information, practical advice and signposting and was delivered by doctors, psychologists or nurses. Interventions leading to fewer suicide attempts in countries where better mental health services exist were based more on psychological theories underpinning suicidal behaviour [24, 25] and psychological techniques to explore motivation for change and safety strategies delivered by trained clinicians or therapists [24, 25]. These differences are consistent with realist evaluation [29] pointing to what works in which circumstances and for whom. In three studies, follow-up contact was over the telephone. This makes interventions more viable and cost-effective when resources are scarce while also allowing for flexibility and improved access to treatment when, for example it might be geographically unavailable [30].

Similar to a previous review of suicide interventions [7], the contribution of the individual components of the interventions is unclear as the interventions were evaluated as a whole. Moreover, it is not clear what the contribution of more frequent contacts is and up until which point these contacts are optimally effective.

One of the four studies was conducted in low and middle-income countries, which has implications for the generalizability of the results to countries with stronger health and social care systems. Treatment as Usual is described in the studies as usual care in clinical practice. This is likely to have varied considerably as the studies were conducted in different countries with different healthcare systems. For example, this consisted of inpatient, day patient and outpatient care in the study by Gysin in Switzerland. However, this is likely to have been considerably less (“as per the norms in the respective EDs”) in the Fleischmann study which was conducted across 5 different low and middle-income countries. This introduces considerable heterogeneity in interpreting the findings.

All of the studies evaluated interventions that were implemented with people after attending the ED, with two interventions explicitly also involving family or significant others [22, 25] Two studies focus on high risk populations, i.e., people in low and middle-income countries [23] and young people [25]. The ED setting is particularly important as a large number of at-risk individuals use emergency services [31]. People who attend the emergency department are at high risk of a further suicide attempt, with studies showing that around 20% re-present within one year [31]. It is estimated that hospitals in England manage over 200,000 episodes of self-harm each year. Many people who attend the ED in a crisis do not attend specialist mental health services for follow-up. Hence, brief ED interventions to reduce suicide risk may be especially useful [32]. Although the identified studies were conducted in the ED, the interventions – as a whole or components of the interventions - could be tested in other treatment settings such as inpatient or outpatient community treatment settings.

### Strengths and limitations

This review used a systematic approach to identify controlled studies of brief psychological interventions for suicidal thoughts and behaviour. It identified the usefulness of brief interventions to address suicidality in the ED. However, and as previously found [11], the evidence base is small. As is commonly found in systematic reviews with a limited evidence base, the included studies were disparate in their design, outcome measurement tools, measurement intervals and types of interventions offered. As they assessed different outcomes at differing time points, a meta-analysis was not appropriate. In addition, one of the four studies was conducted in low and middle-income countries, which has implications for the generalizability of the results. The findings from this study may not be generalizable to higher income countries with stronger health and social care systems. However, the narrative synthesis allowed us to summarise the state of the art across somewhat heterogeneous studies. Detailed information was lacking on specific aspects of the intervention (e.g. length of components and randomisation procedure) in some studies, which restricted the interpretation of the methodological quality. Risk of bias may be reduced if those assessing outcomes are blinded to treatment allocation. However, as some of the outcomes are objective, such as suicide [23], these are not subject to bias. With suicide attempts, there is some element of judgment but, if assessors are blinded, there is less chance of bias. Assessors were blinded in Miller and King but not in Gysin. Suicidal ideation is self-rated and so cannot be blinded. Finally, as they are receiving a psychological intervention, it is not possible to blind participants to treatment allocation.

The review focused on brief interventions that aimed to enhance treatment. While, these interventions often included considerable follow-up contact, we did not include studies solely focusing on follow-up contact. For example, a study in France consisting of a single telephone contact one month after attending the ED was categorized as a follow-up intervention rather than enhancing the index treatment episode/s [33].

## Conclusions

Although there are relatively few studies to date, brief psychological interventions appear to be effective in reducing suicide and suicide attempts. However, it is unclear to what extent the effect is due to specific psychological techniques/components or to more frequent contacts, which should be investigated in future studies. All studies to date have been conducted in the ED. The interventions tested do not appear to reduce suicidal ideation, suggesting that although  patients may still be in considerable distress, the interventions affect change in behaviour, i.e., fewer suicide attempts and suicides, by targeting information and understanding about the suicidal crisis, safety planning for future crises and follow-up contact to monitor and support patients. Early engagement and therapeutic intervention based on psychological theories of suicidal behaviour, sustained in follow-up contacts, may be particularly beneficial.

## Additional files

Additional file 1: Search strategy. Predefined search strategy developed in Ovid MEDLINE(R) 1946 to Present. (DOCX 14 kb)

Additional file 2: Critical Appraisal Skills Programme (CASP) for a Randomised Controlled Trial. CASP for randomised controlled trials checklist. (DOCX 22 kb)

## Abbreviations

ASSIP: Attempted suicide short intervention program
BIC: Brief intervention and contact
CASP: Critical appraisal skills programme
ED: Emergency department
RCT: Randomised controlled trial
SAFTI: Safety assessment and follow-up telephone intervention
TAU: Treatment as usual
TOC: Teen options for change
