# Supplementary material for: Effectiveness of brief psychological interventions for suicidal presentations: a systematic review
Source: BMC Psychiatry. 2018 May 3;18:120. doi: 10.1186/s12888-018-1663-5 (PMC5934886; doi:10.1186/s12888-018-1663-5)
Supplement: Supplementary file 1 — Search strategy. Predefined search strategy developed in Ovid MEDLINE(R) 1946 to Present. (DOCX 14 kb) [file 12888_2018_1663_MOESM1_ESM.docx]

**Additional file 1.** **Search strategy**

Developed in Ovid MEDLINE(R) 1946 to Present.

1 *suicide/ or exp suicidal ideation/ or exp suicide, attempted/

2 thoughts of death.ti,ab.

3 suicid*.ti,ab.

4 self-harm.ti,ab.

5 ending own life.ti,ab.

6 taking own life.ti,ab.

7 1 or 2 or 3 or 4 or 5 or 6

8 exp Clinical Trial/ or double-blind method/ or (clinical trial* or randomized controlled trial or multicenter study).pt. or exp Clinical Trials as Topic/ or ((randomi?ed adj7 trial*) or (controlled adj3 trial*) or (clinical adj2 trial*) or ((single or doubl* or tripl* or treb*) and (blind* or mask*))).ti,ab.

9 exp Case–control Studies/ or Control Groups/ or Matched-Pair Analysis/ or ((case* adj5 control*) or (case adj3 comparison*) or control group*).ti,ab.

10 8 or 9

11 exp Interpersonal Relations/ or exp Communication/ or communicat*.ti,ab.

12 (session* or appointment or meet* or encounter or explor* or clinical formulation or

uncover* or disclos* or express* or inform* or mention* or reveal* or say* or speak* or probe or probing or dialogue or articulat* or contact).ti,ab.

13 (assess* or examin* or talk* or language or elicit* or evok* or ask* or dialogue or convers* or exchange or discus* or discourse or question* or interview* or consult* or interact* or counsel* or session* or appointment or meet* or encounter).ti,ab.

14 (online adj10 suicide).ti,ab.

15 (helpline adj10 suicide).ti,ab.

16 (treatment adj10 suicide).ti,ab.

17 (therapy adj10 suicide).ti,ab.

18 (admit or voice or verbalise or convey or clinical review or therapeutic alliance or relationship or doctor-patient relationship or support*).ti,ab.

19 risk assessment.ti,ab.

20 (verbal or non-verbal).ti,ab.

21 11 or 12 or 13 or 14 or 15 or 16 or 17 or 18 or 19 or 20

22 7 and 10 and 21

We used the following commands specific to the interface:

Adj Words have to appear next to each other

$ Truncation symbol

.ti,ab Restricts the search to title and abstract fields

.tw Restricts the search to title, keywords and abstract fields

EXP Explode the subject heading, to retrieve more specific terms

/ MeSH heading

? Optional wild card character used within, or at the end of, a search term to substitute for one or no characters
