# Supplementary material for: Effectiveness of brief psychological interventions for suicidal presentations: a systematic review
Source: BMC Psychiatry. 2018 May 3;18:120. doi: 10.1186/s12888-018-1663-5 (PMC5934886; doi:10.1186/s12888-018-1663-5)
Supplement: Supplementary file 2 — Critical Appraisal Skills Programme (CASP) for a Randomised Controlled Trial. CASP for randomised controlled trials checklist. (DOCX 22 kb) [file 12888_2018_1663_MOESM2_ESM.docx]

### Additional file 2. Critical Appraisal Skills Programme (CASP) for a Randomised Controlled Trial.

|  | Fleischmann et al. | Gysin-Maillart, et al. | King et al. | Miller et al. |
| --- | --- | --- | --- | --- |
| **1. Did the trial address a clearly focused issue?** | Yes | Yes | Yes | Yes |
| **2. Was the assignment of patients to treatments randomised?** | Yes | Yes | Yes | No |
| **3. Were patients, health workers and study personnel blinded?** | Patients - yes  Health workers –unclear  Assessors - unclear | Patients - no  Health workers - no  Assessors - no | Patients - yes  Health workers –unclear  Assessors - yes | Patients - yes  Health workers - no  Assessors -yes |
| **4. Were the groups similar at the start of the trial?** | Yes | Yes | Unclear | Broadly similar |
| **5. Aside from the experimental intervention, were the groups treated equally?** | Yes | Yes | Yes | Unclear |
| **6. Were all of the patients who entered the trial properly accounted for at its conclusion?** | Yes | Yes | Yes | Yes |
| **7. How large was the treatment effect?** | 90% relative risk reduction for completed suicides | 69% (RR = 0.31)  80% hazard ratio | Large effect for depression  (Cohen's d = 1.07); Moderate for hopelessness; Small for suicidal ideation & alcohol use | 20% relative risk reduction in repeat suicide attempts |
| **8. How precise was the estimate of the treatment effect?** | CI 0.02 to 4.45 | CI 0.12 to 0.80 (relative risk)  CI 12.4 to 13.7 (hazard ratio) | CI not reported | CI 0.63 to 1.02 |
| **9. Can the results be applied in your context? (or to the local population?)** | Yes | Yes | Yes | Yes |
| **10. Were all clinically important outcomes considered?** | Yes | Yes | Yes | Yes |
| **11. Are the benefits worth the harms and costs?** | Yes | Yes | Yes | Yes |
